# A Case of Sporadic Multiple Colonic Polyps in a Young Woman

**DOI:** 10.3390/curroncol30020100

**Published:** 2023-01-17

**Authors:** Seung Ho Sin, Jung Hwan Yoon, Sang Woo Kim, Won Sang Park, Hiun Suk Chae

**Affiliations:** 1 Department of Internal Medicine, Uijeongbu St. Mary’s Hospital, 271, Cheonbo-ro, Uijeongbu-si 11765, Gyeonggi-do, Republic of Korea; 2 Department of Pathology and Functional RNomics Research Center, College of Medicine, The Catholic University of Korea, 222 Banpo-daero, Seocho-gu, Seoul 06591, Republic of Korea

**Keywords:** colon, adenoma, APC gene, mutation

## Abstract

Sporadic colorectal cancer arises from an adenoma. As mutations in the adenomatous polyposis coli (APC) tumor suppressor gene have been frequently detected in colorectal adenomas, the APC gene is considered a gatekeeper in colorectal carcinogenesis. Here, we report a case of sporadic multiple colonic adenomas that were accompanied by an APC-truncating mutation. A 25-year-old Korean woman presented with dozens of incidentally found colonic polyps. There was no family history of colorectal polyposis or colon cancer in her first or second-degree relatives. All the polyps were removed endoscopically at once, and their pathological examination revealed tubular adenoma. Mutational analysis showed a 2-bp deletion mutation at codon 443, which generates a premature stop codon at codon 461 of the APC gene, and Western blot analysis demonstrated both wild-type and truncated APC proteins in adenoma tissue. This study suggests that a single truncating mutation of the APC gene may initiate adenoma formation.

## 1. Introduction

Colorectal cancer is one of the most common cancers and the second leading cause of cancer deaths worldwide [1]. Colonic adenomas are benign epithelial tumors that originate at the tip of the crypts. Colorectal adenomas are known as precursors of colorectal adenocarcinoma; their removal can reduce the rate of colon cancer incidence [2]. Familial adenomatous polyposis (FAP) caused by mutation of the APC tumor suppressor gene accounts for approxiately 1% of all colorectal cancers, and if left untreated, the risk of colorectal cancer in FAP is almost 100% [3,4]. According to the multistep carcinogenesis model of colorectal cancer developed by Fearon and Vogelstein, colorectal cancer begins with the transformation of normal colorectal epithelium to an adenoma, and then progresses through the stepwise accumulation of multiple genetic alterations, subsequently leading to invasive and metastatic tumors [5]. Current studies implicate aberrant Wnt/β-catenin signaling following the inactivation of the APC gene or activating mutations in the β-catenin gene as the initiating event in colonic adenoma development [6,7,8,9]. Complete inactivation of the APC gene accounts for the majority of FAP characterized by profuse colonic polyposis and colorectal carcinoma [10,11]. In addition, inactivating mutations of the gene have been reported in 34–70% of sporadic colorectal cancer patients [12,13,14,15,16]. The majority of the germline APC mutations occur in the first half of the coding region [17], but somatic mutations are clustered in the central region of the open reading frame [18,19].

Interestingly, incidental adenomas have been found in young patients under the age of 40 who do not have strong family histories of colorectal cancer, indicating that they harbor some type of genetic predisposition to developing colorectal cancer [20]. In particular, colorectal cancer in young adults is more aggressive, with a later stage presentation [21]. The criterion that the presence of an adenomatous polyp discovered before age 40 was supposed to trigger an assessment for genetic syndromes was excluded from the revised 2004 Bethesda guidelines [22]. Thus, it is currently unclear if young patients with colorectal adenomas have a higher propensity for colorectal cancer. Herein, we describe a case of sporadic multiple colonic adenomas with an APC truncating mutation in a young patient.

## 2. Case Report

**Figure 1:**
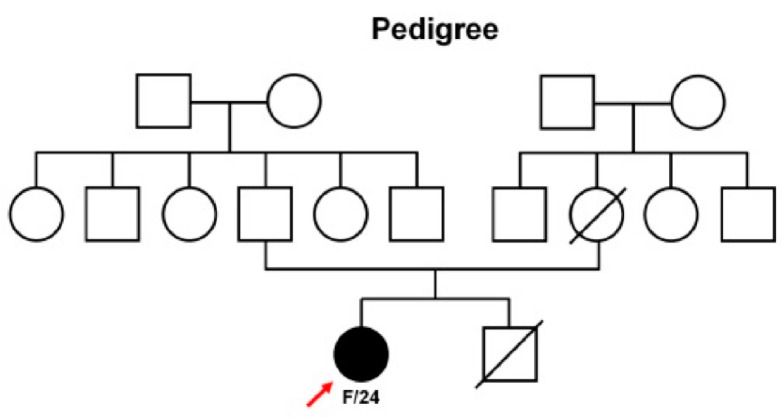
Family pedigree of the patient with colorectal adenomas. The patient is designated with an arrow. Open symbols are unaffected individuals, filled symbols are hemizygous individuals, and symbols with diagonal lines indicate deceased subjects.

**Figure 2:**
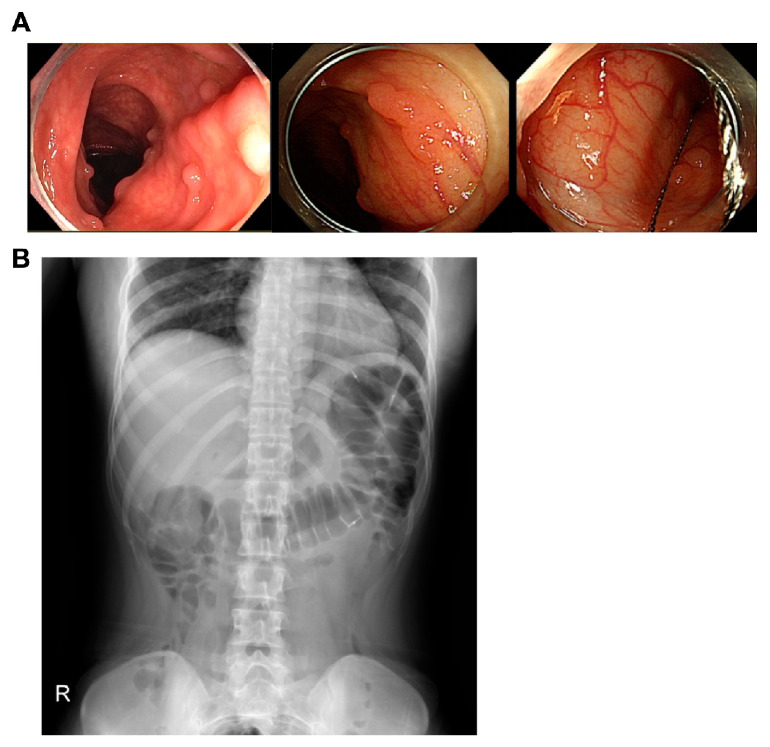
Colonofiberscopy and plain abdominal X-ray of the patient. (**A**) Colonofiberscopy results showing multiple sessile diminutive colon polyps and the cold-snare polypectomy procedure. (**B**) Plain abdominal X-ray after the colonoscopy, showing hemoclips marking the anatomical location of the colon polyps.

A 25-year-old woman was transferred to the Department of Internal Medicine at Uijeongbu St. Mary’s Hospital from a local clinic for the evaluation of colon polyps incidentally found during a medical check-up for employment. She did not have a medical history and appeared generally healthy. There was no family history of colorectal polyposis or colon cancer in her first and second-degree relatives (Figure 1). However, her mother had expired from lung cancer at 43 years of age and her younger brother died at 20 years of age due to an intestinal obstruction of unknown cause. The patient did not complain of gastrointestinal problems. Both the physical examination and laboratory data, including complete blood count and serum carcinoembryonic antigen level, were within the normal limits. Colonofiberscopic examination revealed dozens of diminutive polyps particularly localized in the transverse colon and marked with two hemoclips (Figure 2), which were removed endoscopically with a cold snare (Figure 2). Histologically, all of these polyps were proven to be tubular adenomas. Esophagogastroduodenoscopy showed non-specific abnormalities such as gastric polyps. Endoscopically, there were no visible colon polyps 6 months after polypectomy. Written informed consent was received from the patient in accordance with the Declaration of Helsinki. Samples from the patient were collected after approval by the Ethical Review Board of Uijeongbu St. Mary’s Hospital (IRB No. UC21ZISI0147). 

**Figure 3:**
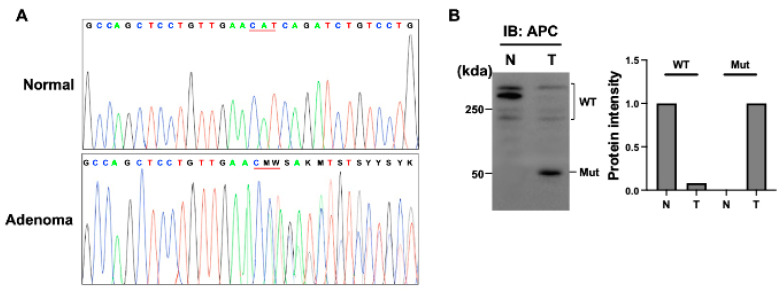
Sequencing and immunoblot results of the APC mutation. (**A**) Sequencing confirmation of the heterozygous deletion mutation in APC. Heterozygous deletion mutation of c.1331-1332del in exon 11 of APC was found using whole-exome sequencing and confirmed via Sanger sequencing analysis. (**B**) Immunoblot of protein extracts from normal (N) and adenoma (T) tissues using APC-specific antibodies.

Whole-exome sequencing of the tubular adenomas and surrounding non-neoplastic mucosa was performed to identify the gene responsible for these polyps. As a result, 248 genes that were somatically mutated in these adenomas were found; of these, seven high impact variants in the ABCA4, MTA3, USP17L20, NIPAL1, APC, NOS1, and FANCM genes were found (Table 1). Particularly, a deleterious truncating mutation in the APC gene was identified, and high-depth amplicon sequencing confirmed the heterozygous deletion mutation at codon 443 (c.1331-1332del) (Figure 3A), which generates a premature stop codon at codon 461, in three adenoma tissues. Western blot analysis detected both wild-type and truncated APC proteins in the adenoma tissue (Figure 3B and Figure S1). Other genes have not previously been known as candidate drivers of colorectal adenoma.

## 3. Discussion

Mutation of the tumor suppressor APC gene, located on chromosome 5q21-22, is the most frequent early genetic event in colorectal carcinogenesis [23]. The gene encodes a protein that affects the Wnt signaling pathway, which functions in cellular processes, including transcription, cell cycle control, migration, differentiation, and apoptosis [24]. Improper ß-catenin accumulation, which is caused by mutation of the APC gene, predisposes patients to develop colon tumors [6]. However, homozygous loss of APC alone is insufficient for nuclear accumulation of ß-catenin, suggesting that initiation of adenoma formation following APC loss occurs independently of ß-catenin, and that ß-catenin nuclear localization promotes adenoma progression to carcinomas [7]. 

The majority of APC gene mutations are deletions, insertions, or nonsense mutations, which result in the formation of a truncated APC protein. APC gene mutations in the mutation cluster region between codons 1286–1513 are associated with allelic loss, whereas tumors with mutations outside this region tend to harbor truncating mutations [4,12,25]. APC mutations that lead to protein truncation were found in 37% of sporadic colorectal carcinoma patients [26]. The finding of an APC mutation in this patient, leading to truncated protein with the complete loss of intact 20-amino acid repeats, which are the critical domains for β-catenin regulation, is a novel APC mutation and suggests that the mutation contributed to adenoma formation by driving the activation of Wnt/β-catenin signaling [12,27]. APC mutations are generally sufficient for colorectal tumors to grow to about 1 cm in diameter, indicating that additional hits are not necessary for these tumors to initiate neoplastic growth [28,29]. In particular, a single truncated APC allele was sufficient to initiate early molecular tumorigenic activity [30]. It was previously suggested that a stable truncated APC protein could inactivate APC transcribed from the wild-type allele in a dominant-negative fashion [31]. Shorter APC protein can inactivate full-length wild-type APC protein by homodimerization at the amino terminus [32]. Here, we found a novel APC truncating mutation, generating a premature stop codon, which resulted in the complete loss of 20-amino acid repeats and the c-terminal sequence in sporadic colonic adenoma. Although the functional sequelae of the APC mutation remain to be determined, the truncating APC mutation may contribute to the formation of these adenomas. 

Colonofiberscopy is regarded as the best method to identify and remove adenomas, which subsequently decreases the risk of colorectal cancer [33]. The incidence of metachronous colorectal neoplasia following polypectomy in people younger than 50 years old does not appear to be greater than in older adults [34]. As we found the APC mutation in multiple adenomas in a young patient, close follow-up and the identification of metachronous polyps in colonoscopy in parallel with surveillance for APC mutation-associated diseases, such as stomach and small intestinal adenomas, were needed. In addition, the genetic predisposition to these adenomas should be examined in young patients with incidental or sporadic multiple adenomas. 

Our study had some limitations. Samples from the patient’s family members were not available. Notably, approximately 25% of FAP patients do not have any family history of disease and harbor a de novo APC mutation [35,36,37]. Thus, we could not completely exclude the possibility that the patient had germline mutation or de novo mutation. In addition, we did not confirm the mutation in the APC gene in all of the polyps due to the small size of the polyps, and we could not rule out the possibility that other uncharacterized driver genes exist. Further studies are required to determine the implication of the other genes in sporadic multiple colonic polyps.

## 4. Conclusions

Here, we present sporadic multiple colonic tubular adenomas confined to the distal transverse colon in 25-year-old woman. Interestingly, a novel APC truncating mutation was detected in these adenomas. Thus, the case emphasizes the need to examine the genetic predisposition to these adenomas in young patients with incidental or sporadic multiple adenomas. Clinically, the patient should be closely followed up for colorectal adenoma recurrence and cancer development.

## Figures and Tables

**Table 1:** Seven High Impact Variants in the Tubular Adenomas compared to Surrounding Non-neoplastic Mucosa.

Chrom	Pos	Ref	Alt	Effect	Gene	Feature ID	HGVS.c
Chr1	94,473,263	TGCCGGCACCATTCA	T	Frameshift _variant	ABCA4	NM_000350.3	c.5918_5931delTGAATGGTGCCGG
Chr2	42,936,061	G	GC	Frameshift _variant	MTA3	NM_001330442.2	c.1352dupC
Chr4	9,270,328	G	A	Stop _gained	USP17L20	NM_001256861.1.3	c.984G>A
Chr4	48,037,939	AGTGGTATGGCATGACAGCTG	A	Frameshift _variant	NIPAL1	NM_207330.3	c.985_1004delTGGTATGGCATGAC
Chr5	112,157,610	CAT	C	Frameshift _variant	APC	NM_001354896.2	c.1331_1332delAT
Chr12	117,685,302	G	A	Stop _gained	NOS1	NM_001204218.1	c.2776C>T
Chr14	45,605,385	G	T	Stop _gained	FANCM	NM_020937.4	c.151G>T

Chrom, chromosome; Pos, position; Ref, reference sequence; Alt, Alteration sequence; HGVS, Human Genome Variation Society; del, deletion; dup, duplication.

## Data Availability

The data presented in this study are available on request from the corresponding author. The data are not publicly available due to privacy of the patient.

## Supplementary Materials

The following supporting information can be downloaded at: https://www.mdpi.com/article/10.3390/curroncol30020100/s1, Figure S1: Original western blots for Figure 3.
