# Supplementary figures and images for: A Case of Sporadic Multiple Colonic Polyps in a Young Woman
Source: Curr Oncol. 2023 Jan 17;30(2):1293–9. doi: 10.3390/curroncol30020100 (PMC9955090; doi:10.3390/curroncol30020100)

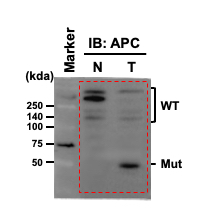

Supplement: Supplementary file 1 [file curroncol-30-00100-s001.zip › curroncol-2117502-supplementary.jpg]
